# The Happiness, Hardiness, and Humor Styles of Students with a Bachelor’s Degree in Sport Sciences

**DOI:** 10.3390/bs8090082

**Published:** 2018-09-12

**Authors:** Pınar Yaprak, Mehmet Güçlü, Tebessüm Ayyildiz Durhan

**Affiliations:** Department of Recreation, Faculty of Sport Sciences, Gazi University, Ankara 06330, Turkey; mguclun@gmail.com (M.G.); tebessum@gazi.edu.tr (T.A.D.)

**Keywords:** happiness, humor, positive psychology, hardiness, resilience, sports, wellbeing

## Abstract

Happiness, psychological hardiness, and humor (“the 3Hs”) are useful ways of assessing resilience to stress in positive psychology. The literature analyzing their confluence regarding sports is scarce. This study aims to analyze the participants’ 3H levels and the relationships between those levels and specific variables. The Psychological Hardiness Scale (Psikolojik Dayaniklilik Olcegi, PDO), the Oxford Happiness Questionnaire Short Form (OHQ-SF), and the Humor Styles Questionnaire (HSQ) were used. Students in pedagogical formation training during the 2016–2017 fall semester have participated in the study (*N* = 211). Significant differences were found regarding the levels of psychological hardiness in relation to gender, type of sport, and years of participation, and, in humor types regarding the variables of gender, age, residence and perceived income. There were significant differences in all instruments regarding gender. Negative relationships were found between the “aggressive humor” and “challenge” and “self-commitment” sub-dimensions of PDO, while the relationships were positive between “self-enhancing humor” and PDO and OHQ-SF, and between “affiliative humor” style and PDO and OHQ-SF. This study enhances the positive socio-psychological account in the literature by incorporating the issues of 3H and provides an understanding of particularities that may help improve the practice of relevant experts and individuals.

## 1. Introduction

Happiness, psychological hardiness, and humor (“the 3Hs”) are often pointed to as useful ways of assessing resilience to stress when undertaking professional tasks [1]. They are associated with positive psychology, usually as accompaniments to coping mechanisms. Positive psychology criticizes traditional psychology for having “devoted very little attention to the study of wellness and the ‘positive’ aspects of life, such as resilience, character strengths and well-being” and having “focused too heavily on pathology” and problems such as depression, anxiety, and post-traumatic stress disorder [2]. The field of positive psychology is about valued subjective experience (such as wellbeing and happiness), positive individual traits (such as the capacity for love, courage, interpersonal skill, forgiveness, and talent) and “the civic virtues and the institutions that move individuals toward better citizenship” (such as responsibility, nurturance, tolerance, and work ethic) [3]. The positive psychology concept has been used in sports regarding positive behaviors, environments, outcomes, and organizational psychology [4,5]. This approach to sport studies preexisted the notion of positive psychology, with considerable attention paid to “what it takes to be a great athlete and how to facilitate performance” [4]. The approach is embraced not only in the pursuit of individual athletic excellence in elite or high-performance sports but also regarding individual and organizational developments in sports organizations as sites of psychological strengths and capabilities [4,5].

### 1.1. Humor

Humor is one of the three main dimensions in the current study. The positive psychology perspective views humor “as a personal quality that promotes resilience and well-being by means of cognitive reappraisal of stressful events” [6,7]. The literature (sporting or non-sporting research) suggests that the particular usage of humor (such as positive humor) may contribute to healthy communication, relationships, quality of life, positive organizational culture and physical and psychological health in general [8,9,10,11,12]. On the other hand, negative usages of humor (such as to humiliate or exclude someone) may cause harm to one’s wellbeing or status; therefore, it is important to understand the particularities in relation to humor to foster positive usage and prevent psychological violence [8,9,10,11,12].

### 1.2. Resilience (Psychological Hardiness)

The positive use of humor is considered a personal trait that promotes resilience and wellbeing. Likewise, resilience or psychological hardiness (another dimension in the current study) has been examined as an internal resource and personality trait reducing the negative effects of stressful life events on physical and mental health [13,14,15,16,17]. There are other terms such as mental toughness and psychological resilience used in the literature to refer to a similar theoretical argument. Challenge, control, and commitment were the three most applied dimensions when testing mental toughness, psychological hardiness, or resilience [13,16].

There is a notable co-existing issue in relation to sports and resilience or psychological hardiness: (a) the ability of psychological hardiness or resilience to mitigate the negative impact of highly demanding sporting performance environment on wellbeing and (b) psychological resilience as a positive psychology developed through sports participation to facilitate improved performance [17]. The highly demanding performance environment of sporting tasks (including contexts of elite or amateur athletes, trainers, physical education teachers or other sporting employees) is considered to require physical and psychological resilience [17,18].

Although resilience or psychological hardiness is especially important in rescue work occupations, as these employees are more frequently exposed to stressful and potentially traumatic events [16], few relevant examinations in relation to sports are found in the literature, especially regarding athletes engaged in high-performance sports [17]. Hence, it is important to remember that sport sciences graduates have likely been employed in rescue work occupations and engaged in high-performance sports for some time.

### 1.3. The “3Hs”: Humor, Psychological Hardiness, and Happiness

The relationships among the 3Hs have been often found to be positive, while a few studies have referred to negative relationships between some elements of humor and resilience [7]. In addition to their interactions, attention should be paid to broader issues regarding the society or environment that a person interacts with. For example, professional development and the quality of teacher education or training programs are important for understanding the current state of sporting employees or athletes since such elements of social environments are connected to components of psychological wellbeing such as self-acceptance, a positive relationship with others, autonomy, environmental mastery, and personal growth [18,19]. Thus, it is important to increase knowledge regarding the relationships between the 3Hs and development on and beyond the sports field.

Moreover, from a resiliency perspective, “several positive character strengths, such as kindness, humor, leadership, love and social intelligence, all showed significant increases in growth” following major traumatic experiences, such as “a life-threatening accident, attack, or illness” [7,20]. Concordantly, the literature suggests that the social and “numerous psychological factors (relating to a positive personality, motivation, confidence, focus, and perceived social support) interact to influence the stress‒resilience‒performance relationship” [17]. Likewise, physical activity has been associated with higher levels of happiness, though the association with happiness was domain-specific [21].

Furthermore, resiliency-based approaches to humor in literature are limited due to their sole focus on humor as a positive attribute. Thus, the humor styles model, which acknowledges both positive and negative aspects of humor, is suggested to be used within a resiliency perspective [7]. However, more research is needed to understand the particularities in relation to the 3Hs regarding the aforementioned complex elements and their interactions. The literature on these complexities is growing. Such attempts to shed light on more aspects can offer significant contributions to the literature [8].

In line with some of these factors and the importance given to their co-existence and interaction, we aimed to analyze the (a) 3H levels of students who have a bachelor’s degree in sport sciences and (b) examine the relationships between those levels and specific variables, therefore providing an understanding of some of the particularities regarding the 3H scores of sport sciences graduates. Overall, the literature on the interactions between the elements of 3H is scarce, not only for sports but also in general. Sports studies, especially the sports psychology field, have played an important role in helping “the field of general psychology move into a millennium of positive psychology” [4]. However, there is a need to expand our relevant understanding to identify the psychosocial factors that underpin organizational excellence in those sporting social actors (such as athletes, physical education (PE) teachers, coaches, managers, service providers, administrators, and policy makers) who operate within these fields on a day-to-day basis [5]. This study aims to increase knowledge that will serve such actors well in this domain. Increasing the competitiveness of PE can be achieved by acquisition of complex and interdisciplinary professional knowledge and skills, optimizing the attitudes and behavior of teachers and diversifying proactive motivations in accordance with the modern didactic requirements of physical activities [22,23,24]. In addition to the pursuit of improved skills through examining these sporting contexts of positive psychology, the current study is important in terms of aiming to contribute to knowledge in “pursuit of employee psychological well-being beyond achieving high job performance” [18].

## 2. Materials and Methods

### 2.1. Design and Participants

Quantitative methods and a general survey model were used in the study. A total of 211 students who have a bachelor’s degree in sport sciences and were in pedagogical formation training during the data collection participated in this study.

There was an approximately equal number of men (*n* = 105) and women (*n* = 106) participating in this study. Most of the students perceived their income as medium (86.3%, *n* = 182) and lived within metropolitan municipalities, megacities, and cities (*N* = 185), while fewer participants lived in towns (*n* = 26). Moreover, most students were not national athletes (81.5%, *N* = 172). However, approximately half the students were trained in an exercise sciences department (41.7%), where there have usually been more students with a high-performance sporting background compared to the sports management and recreation departments at bachelor’s level in Turkey. Half of the students had 10 years or more of sports participation; the participation years and number of respondents decreased together (Table 1).

While 31.8% of the respondents were engaged in both individual and team sports, slightly more than half (54.5%) of the participants were engaged in team sports only, and fewer participants were engaged in individual sports only (13.7%). Results of correlational analyses regarding all these sporting and demographical backgrounds are shared in the following main sections.

### 2.2. The Instruments

The Psychological Hardiness Scale (Psikolojik Dayaniklilik Olcegi, PDO) [25], the Oxford Happiness Questionnaire Short Form (OHQ-SF) [26], and the Humor Styles Questionnaire (HSQ) [27] were used together with an information form on sporting and socio-demographic background to determine the 3H levels of the participants and the relationships between the 3H levels and specific variables. The Cronbach’s alpha coefficients of the measurement tools were found to be 0.84, 0.74, and 0.76, respectively, in the current study.

The questions on sporting background were to provide data on a participant’s (sports) departments, length and type of sports participation, and whether they were national athletes or not, while the questions on socio-demographic background were to collect information on a participant’s gender, parents’ education, perceived income, and residential area (Table 1).

The PDO is one of the three instruments used in this study. This instrument “was developed to determine the extent of psychological hardiness” or resilience [13]. It is a 21-item self-reported questionnaire that consists of three sub-dimensions (commitment, control, and challenge). The items are rated on a five-point Likert type (1: Absolutely agree to 5: Absolutely disagree) [13].

The OHQ-SF is another instrument used extensively and internationally. The Turkish version [23] of the OHQ-SF [25] is a seven-item self-reported measure of happiness rated on a five-point Likert type (1: Absolutely agree to 5: Absolutely disagree). The OHQ-SF and HSQ instruments were recommended as a reliable and valid measure among Turkish university students [25,26].

Finally, the Turkish version [26] of HSQ, as in the original instrument [28], assesses the following four dimensions of individual differences in uses of humor (the first two are positive, while the last two are negative dimensions): (1) “relatively benign uses of humor to enhance the self (Self-enhancing)” and (2) “to enhance one’s relationships with others (Affiliative)”, (3) “use of humor to enhance the self at the expense of others (Aggressive)”, and (4) “use of humor to enhance relationships at the expense of self (Self-defeating)” [26,28].

### 2.3. Procedure

The idea of this research stemmed from a comprehensive study on humor and sports in which the perspectives of grounded theory methodology were adopted by the corresponding author. The second author has suggested using humor in relation to the psychological instruments. Therefore, the current article was organized by the research team (the aforementioned authors) with suggestions from the interdisciplinary experts who gave their permission to use the relevant scales.

The possible instruments in relation to humor were determined with the suggestions of the field specialists. A discussion panel was made up of field experts, including the scholars who developed or used the relevant instruments. Both these scholars and the literature [7] have suggested the use of either two or three instruments or their consideration together.

The proposal of this research was submitted for ethical review, guidance, and approval, and the approvals were provided by the institutional review board of Gazi University (issue 92236698-903.99-, decision number 321), in addition to the reviews and approvals by the field experts. The students included in the study were sport sciences graduates who volunteered to participate and were able to provide informed consent. The students were chosen in accordance with the convenience sample. They were in pedagogical formation training at the university during the end of fall semester of the 2016–2017 academic year and were easily accessible. Overall, this research was carried out in accordance with the ethical principles of the Helsinki Declaration. This article was presented at and published in the abstract book of the 15th International Sport Sciences Congress in Antalya, Turkey, in 2017 [1].

### 2.4. Analysis of Data

Descriptive statistics were used to summarize the sporting and socio-demographic backgrounds of the sample (Table 1) and the mean scores and basic features of the 3H instruments (Table 2). The *t*-test and one-way ANOVA were used to compare the 3H levels between (such as gender) or among (such as the type of sports and residence) different sporting and socio-demographic backgrounds, respectively. The Pearson correlation coefficient was used to measure the correlations between the scales, including their sub-dimensions. Overall, the descriptive, correlational (*t*-test, one-way ANOVA, and Pearson correlation coefficient), and reliability (Cronbach’s alpha) analyses were performed using the SPSS software package.

## 3. Results

The results with significant differences are provided in the tables below. First of all, there were no significant differences in the levels of PDO, OHQ-SF, HSQ, and respective sub-dimensions according to participants’ departments, being a national athlete or not, and their parents’ educational status.

Secondly, the mean scores of 3H instruments were relatively high in the current study (Table 2). In addition, compared to the negative ones (“aggressive and self-defeating”), participants scored higher in the positive humor (“affiliative and self-enhancing”) sub-dimensions.

When comparing males and females, significant differences were found in PDO total scores (*p* < 0.00) as well as in the “challenge” (*p* < 0.05) and “commitment” (*p* < 0.00) PDO sub-dimensions (Table 3). Moreover, women scored higher than men in all PDO items. In addition, women scored significantly higher than men in OHQ-SF (*p* < 0.00). However, a significant difference was only found in “affiliative” humor styles (*p* < 0.05); women scored higher in both positive humor styles (affiliative and self-enhancing) and men scored higher in both negative ones (aggressive and self-defeating) (Table 3).

While there were no significant differences between the age groups regarding the PDO and OHQ-SF, significant differences were found in the HSQ total score, “self-enhancing”, “aggressive”, and “self-defeating” humor styles (Table 4). According to these significant differences, the 23 and under age group scored the highest and the 32 and over age group scored the lowest (Table 4). The humor style scores decrease with age with respect to the total HSQ scores. However, in contrast to the total scores, the oldest age group (>32) has the highest score in “affiliative” humor style, although, the difference is not significant. Moreover, there is an increase in the 28–32 age group in “aggressive” humor; on the other hand, this group has the lowest score in “affiliative” humor (Table 4).

A significant difference was found regarding the PDO sub-dimension “commitment” when comparing the number of years respondents had participated in sports (*p* < 0.05). The “commitment” sub-dimension score was the highest in the group that participated in sports for “1 to 5 years”, while the participants who responded “6 to 9 years” scored lowest and relatively similar to the group participated in sports for “10 years or more” (Table 5).

Similar to the age groups, there were no significant differences among the residential groups regarding PDO and OHQ-SF, while significant differences were found in HSQ total scores and “affiliative, aggressive, and self-defeating” humor styles (Table 6). The participants who live in megacities had significantly higher HSQ total scores and “affiliative” humor style scores than those who live in metropolitan municipalities or towns. The participants who live in towns had significantly higher scores of “aggressive” humor style compared to respondents who live in cities (Table 6).

The PDO and OHQ-SF scores did not differ significantly, while significant differences were found in “aggressive” humor styles with respect to perceived income (Table 7). The students who perceived their income as “low” had significantly lower aggressive humor style scores than the students perceived their income as “high.” The students who perceived their income as “high” had significantly higher aggressive humor style scores than the students who perceived their income as “medium.” According to these results, the higher the income they perceived, the more aggressive humor style they had (Table 7).

When comparing the levels of PDO, OHQ-SF, HSQ, and respective sub-dimensions between the respondents who participated in different types (individual, team, or both) of sports, significant differences were found in PDO total scores and PDO sub-dimensions of “challenge” and “commitment” (Table 8). According to the PDO total scores, the respondents with individual sports participation scored highest, while the students with team sports participation scored lowest. The results regarding the “challenge” and “commitment” sub-dimensions of PDO were similar to these results regarding PDO total score; the respondents with individual sports participation scored highest, while the students with team sports participation scored lowest (Table 8).

**Figure 1:**
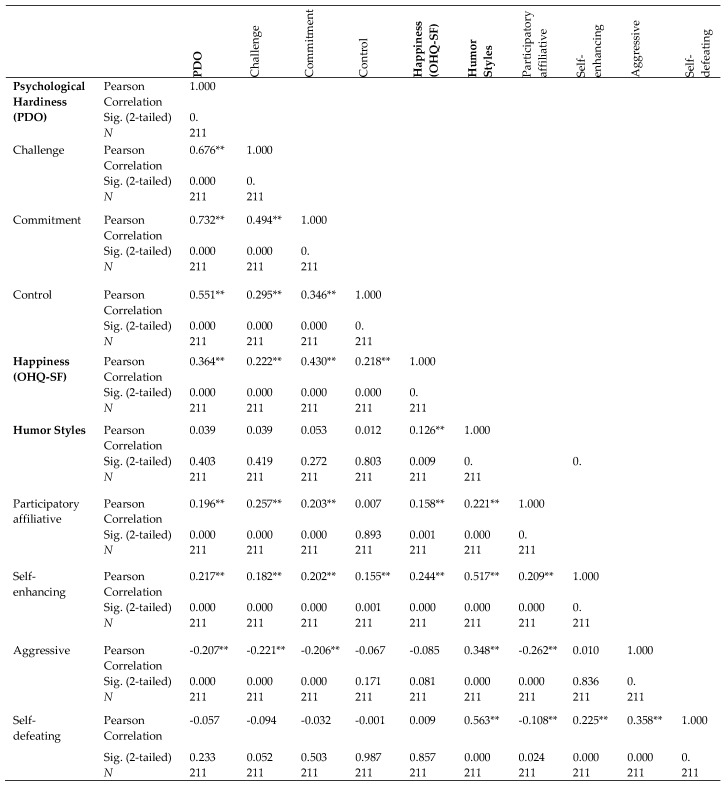
Correlations between instruments and sub-dimensions. ** Weak (0.20–0.39), moderate (0.40–0.69) or strong (0.70–0.89) relationship based on the Alpar (2014) qualification regarding correlation coefficient (Table A1 in [29]). Sig.: 2-tailed test *p*-value; *N*: Total number of students who participated in the study.

Based on the Alpar (2010) qualification regarding correlation coefficient ([29], Table A1), there was a significant very low-level positive relationship between the HSQ and OHQ-SF. In addition, there were a low-level (with “challenge” and “control”) and moderate (with commitment”) positive significant correlations between the OHQ-SF and PDO including the respective sub-dimensions (Figure 1).

There was a low-level positive significant relationship between “affiliative” humor and PDO sub-dimensions except for the “control” sub-dimension of PDO. The relationship between the “affiliative” humor and OHQ-SF was significant but very low (Figure 1).

Moreover, there were very low-level positive significant relationships between the “self-enhancing” sub-dimension of HSQ and the “challenge and control” sub-dimensions of PDO a low-level positive relationship between the “self-enhancing” and “commitment” PDO sub-dimensions, and between the “self-enhancing” sub-dimension and OHQ-SF (Figure 1). On the other hand, the relationships were low-level and negative between the “aggressive” humor and the PDO sub-dimensions “challenge” and “commitment”. In addition, there was a low-level negative relationship between “aggressive” humor and “affiliative” humor, while the relationship was very low and negative between the “self-defeating” and “affiliative” humor styles. Figure 1 provides more details on the relationships.

## 4. Discussion

### 4.1. Relationships Regarding the 3Hs

A vast literature supports the positive relationships between psychological resilience, humor, happiness, and sports participation. The relevant research using similar scales and different combinations of these dimensions point out some common aspects. For instance, psychological hardiness and humor influence wellbeing by reducing the perception of stressful events as threatening and enabling the use of effective coping strategies [7,13,26]. Along with happiness, psychosocial variables such as positive social relationships, self-esteem, self-efficacy, self-mastery, and optimism have also consistently been associated with resilience, better health, and reduced distress [30]. Moreover, “happiness is associated with physical activity participation across multiple countries” [21]. Furthermore, the literature suggests that extroverts are more likely to enjoy and take part in social activities and sports (especially team sports), use humor, seek recreation, and in turn, have increased tendencies for happiness [31,32,33,34,35,36]. However, in another relevant study, introverts enjoyed sports more compared to other social activities that extroverts enjoy [31]. This marks sports as being or having the potential to be a common cultural activity that is more likely to be enjoyed by diverse people and linked with happiness and resilience.

In the current study, all respondents had sports participation background and some of the results were relatively in line with the abovementioned literature. The respondents’ scores were relatively high and there were weak or moderate but positive significant correlations between the PDO sub-dimensions and OHQ-SF. In addition, there was a very low-level but positive significant relationship between the HSQ and OHQ-SF. The significant relationships were very low-level and positive between the “affiliative” humor style and the OHO-SF, and low-level between the “challenge and commitment” sub-dimensions of PDO, which corroborated previous studies that found a positive relationship, especially between the “affiliative” humor style and happiness [28,37].

On the other hand, low-level negative relationships were found between the “aggressive” humor sub-dimension of HSQ and the “challenge” and “self-commitment” sub-dimensions of PDO. Moreover, the negative relationship was significant and low-level between the “aggressive” and “affiliative” humor styles, while it was not significant but still negative between the “aggressive” humor and OHQ-SF and “control” sub-dimension of PDO. In other words, while aggressive humor was positively associated with overall HSQ (revealing a low-level positive significant relationship) in the current study, it also came with less “affiliative” humor style, “challenge” and “self-commitment” (a negative relationship with these sub-dimensions). These results could contribute to the discussions on the interpretation of higher resilience (more in women and persons participating in individual sports and with “affiliative” humor style) and humor of any kind as “healthy, positive, better, happier” since the experiences in the process and output are multifaceted, subjective, or not definite regarding their scientific, personal and societal perception, and interpretation. These results were also consistent with some of the complex explanations on resilience, wellbeing, and humor in the literature suggesting that there are also negative associations between hardiness and mental health outcomes [38]—the processes relating humor use to cognitive appraisals and performance may vary dramatically and may support some aspects of a resiliency model, but not others [7,39]. While “humor use can promote distancing from the sources of stress”, greater use of humor was linked with “more external attributions for failure on a bogus intelligence test” and those who “used humor also spent less time and performed poorer on a subsequent test” [7,40]. In addition, the higher levels of commitment athletes exhibited, the less likely they were to use humor and behavioral disengagement coping strategies [39]. It was also found that “athletes with higher confidence levels in their ability were less likely to use self-blame as a coping strategy” [39]. The participants’ total scores were higher in positive humor sub-dimensions than in negative ones in the current study. However, the overall results and literature revealed the complexity regarding not only the use of negative humor but also the use of positive humor, especially “aggressive, self-enhancing and self-defeating” humor. Their negative relationship with resilience is a recognized state in the literature that can provide more insights into wellbeing or explanations to detect some negative, reduced or adverse performance results. Saying that the studies found a positive relationship between positive humor and coping strategies of teachers, and pointed out positive humor as a “healthy” coping mechanism [8].

The mean scores of all 3H instruments were found to be relatively high. In addition, positive humor total scores were higher than negative ones (Table 2). However, higher PDO sub-dimension and total OHQ-SF and PDO scores refer to higher happiness and psychological resilience [13]. Thus, the humor aspect, especially, was distinct, providing results for positive and negative humor types as well as adverse meanings revealed through their correlation with other dimensions, as discussed above. Moreover, of the six sporting (2) and socio-demographic (4) aspect, the gender was the only common variable that differed significantly in all instruments in the current study (Table 9). Furthermore, the significant results in humor styles included more distinctive aspects. Overall, significant differences in the levels of psychological hardiness were found in relation to gender, type of sports, and the number of years that the respondents had participated in these sports (Table 9). On the other hand, overall significant differences in the humor style scores were in relation to the variables of gender, age, residence and perceived income (*p* < 0.05) (Table 9).

### 4.2. Gender, Age, Perceived Income, and Residence

There was no indication of gender forming a significant difference in many studies on resilience, humor, and happiness, even though gender is one dimension always considered in their methods. Thus, there are still many aspects to reveal regarding gender issues. The results in some of the studies on happiness (with university students from diverse departments) [25,31], humor (with lecturers at the Schools of Physical Education and Sport [12] and resilience (with elite cyclists) [41] illustrated no difference regarding gender.

However, in our sporting research population, women outperformed men since they scored significantly higher than men in OHQ-SF as well as in all PDO items. Moreover, women’s higher scores in positive humor styles (affiliative and self-enhancing) and men’s higher scores in negative ones (aggressive and self-defeating) in HSQ confirmed this. Although the differences in most HSQ sub-dimensions (other than the “affiliative” humor style) were not significant, the features of the instrument enable some interpretation. The instrument includes four humor styles that people use in daily life and that researchers usually distinguish between: “participatory or affiliative” and “self-enhancing” humor styles are positive or beneficial to the self or others, while “aggressive” and “self-defeating” humor styles are negative or detrimental to the self or others [26,37]. Results similar to those of the current study were found in research with high school [42] and university [43] students in Turkey. In these studies, the male students’ “aggressive” and “self-defeating” (negative) humor scores were higher than the scores of female students. In addition, many other international studies indicate that males frequently use the aggressive humor style [44,45,46,47]; according to a study with Chinese junior high school students, this was because males tend to exhibit less empathy than females [44]. Unlike the studies representing the significant difference, the humor styles scores of men and women in the current study were insignificant. This was probably because women with a sporting major exhibited more negative humor or men with a sporting major exhibited less negative humor. Previous studies that did not refer to gender differences and/or provide comparable statistics require further examination and reveal a need for comparable future studies.

However, some results were highlighted in the qualitative literature regarding gender as well as age. A study reporting on responses to “seeing a man riding a unicycle” suggested that women did not make aggressively humorous remarks but had warm, appreciative, and supportive responses with a concern for safety instead, while the “male joke” with repetitive and irritating content, and offensive intent was observed by male and female unicyclists in many parts of the world [47].

Moreover, adult males showed aggressive and a stereotyped humorous response that became less frequent in elderly men [47]. Furthermore, significantly higher scores were found in the aggressive humor styles of research assistants compared with lecturers, assistants, and associate professors and professors; in addition, professors’ life satisfaction levels together with some of their “emotional intelligence” scores (such as coping with stress, interpersonal relationships, and adjustment) were higher than research assistants’ [48]. Another study [49] found a relatively similar tendency regarding the humor type and age; while teachers’ perception of the humor type of the school principal did not differ according to many other variables, it differed significantly according to the teachers’ ages (the oldest group scored higher). While the highest educational leadership scores were given to principals with the generative humor type, the lowest scores were given to the principals with a non-humorous style [49]. In addition, a study of Portuguese athletes highlighted “the role of maturity and experience in the use of more functional and adaptive coping strategies, supporting the developmental and age differences hypothesis in the use of coping” [50].

In the current study, too, as noted, there were significant differences regarding age as well as perceived income and residence in relation to the humor aspect only, whereas their correlations were not significant in relation to the resilience and happiness dimensions. In particular, significant differences in “self-enhancing,” “aggressive,” and “self-defeating” humor styles were relatively similar to the literature [47,48,49]; these humor style scores (together with the total score) usually decreased as age increased. In contrast to this tendency (to decrease with age) in total score, the oldest age group (>32) had the highest score in “affiliative” humor style, which was also in line with the literature [47,48,49,50]. Moreover, there was an increase in the 28–32 age group in “aggressive” humor style; on the other hand, while this group had the lowest score in “affiliative” humor style (Table 4). These results indicate that although the “positive” use of humor was more likely to occur in later age, such adjustments have not been linear. The broader psycho-physiological, sociocultural, and economic factors linked with certain age groups and contexts may provide explanations for these changing results.

For example, an anticipation of coming conflict, such as competitions or in social interactions, can be related to aggression. Moreover, the 28–32 age group may be more critical in terms of stress factors such as career uncertainties. Furthermore, a weak but positive relationship was found between testosterone and human aggression; testosterone among young adults was high but decreased around middle age, which was correlated with physically aggressive behavior in males [51,52]. In addition, the level of testosterone increases when you win, and decreases when you lose in sports [51,53,54]; sporting individuals are inevitably exposed to such physical experiences or memories. However, social studies in sport and violence tend to situate arguments on male aggression away from the on “male nature” in order to point out the greater role of social constructs [55,56]. As a social behavior, aggressive behavior is “a product of predisposing personal factors and precipitating situational factors.” For instance, encoded social cognitions are influential, including schemas about the world and normative beliefs about what is appropriate to interact with situational primes to determine behavior [57]. Accordingly, higher use of aggressive humor regarding certain residential areas and income levels in the current study provided even more evidence for this argument.

Similar to the age groups, there were no significant differences among the residential groups regarding the PDO and OHQ-SF, while significant differences were found in HSQ total scores, “affiliative, aggressive, and self-defeating” humor styles scores (Table 5). The participants who live in megacities had significantly higher HSQ total scores and “affiliative” humor style scores than participants who live in towns or metropolitan municipalities. Students who indicated their residence as a megacity may live around their university, which has a central location in the capital city. Metropolitan municipalities are locations where surrounding residential areas such as towns and villages were integrated with a city or megacity in recent years. Thus, their results were expected to represent similarities to the results from town, as much as from (mega)cities. The participants who live in towns additionally differed in terms of their significantly higher scores of “aggressive” humor style compared to the respondents who live in a city (Table 5). Do aggressive humor affiliations of respondents who live in town result from the number of stress factors (such as the campus not being as accessible) or the socialization (acceptance of “aggressive” humor may be higher in towns)? Does the “affiliative” humor of respondents who live in megacities result from the number of factors providing relief in life (such as more accessible living may be possible and different social interactions may be flourishing their perspectives)? There is a need for further research to answer such questions.

Moreover, significant differences were found, especially in aggressive humor styles, with respect to the perceived income. The higher income they perceived, the higher the score they had for aggressive humor (Table 6). Studies on humor found that socioeconomic status was necessary for subjective wellbeing but not enough on its own. The effects of income on subjective wellbeing are not simple and linear; for example, rising material desires, stressors such as longer work hours, and higher expectations for achievement were among the negative associations with income [43,58].

All in all, alternative categorizations of results are possible which may help varying interpretations. For example, aggressive humor was associated with males, <23 and 28–32 age group, less resilience (self-commitment and challenge), higher income and living in town. On the other hand, “affiliative” humor was associated with females, >32 age group, living in megacity and resilience (“challenge and self-commitment”) positively.

### 4.3. Physical Activity Participation

In the current study, those who had participated in sports for the shortest time (1‒5 years) scored highest in the “commitment” sub-dimension of the PDO, while the participants who responded “6 to 9 years” scored the lowest, relatively similar to the group that had participated in sports for “10 years or more” (Table 4). Although the literature usually did not directly provide information about either the number of years of participation in sports or the relationship with resilience, their age-related results in relation to sports, happiness, humor, or resilience are partly in line with the current study, as indicated in the discussion.

Physical activity participation is associated with happiness in many studies and multiple countries [21,31,32,33,34,35,36]. Moreover, “athletes from individual sports reported higher levels of worry, somatic anxiety, threat perception, and a greater use of venting of emotions,” while “athletes from team sports reported a greater use of humor and substance abuse” [50]. Parallel to these results, the respondents with individual sports participation scored highest in total PDO as well as in the “challenge” and “commitment” sub-dimensions of PDO (similar to women’s results compared to men) in the current study, while the students with team sports participation scored lowest (Table 7). The “challenge” and “commitment” sub-dimensions were significantly and negatively related to “aggressive” humor style, and “aggressive” humor style was associated with the number of variables listed at the end of the previous sub-heading. This result is important in terms of respondents’ possible current or future roles in sports fields as employees or employers. Resilience, hope, and optimism, as components of organizational psychological capital, promote organizational commitment and job satisfaction [59,60].

Studies pointed to many stressors related to the social interactions in the context of the team environment, thus further examination of “the relationship between athletes’ cognitive appraisal processes and different coping strategies in diverse individual and team sports” was suggested [50,59]. Furthermore, the significant difference was not so high in the use of humor or in happiness regarding the type of sports in the current study; it is likely that respondents were sport sciences graduates and have all had both team and individual sports experience.

Furthermore, studies pointed out the possible relationships between team sports, extroversion, and happiness [31,32,33], as well as the greater consumption of alcohol or drugs by team sports athletes as team sports generate additional opportunities for social interaction [50]. These results provide more evidence on the complexity of the 3Hs, socio-demographic and sporting dimensions; more specifically, of the resilience aspect in relation to happiness and humor types [7,38,39].

This study expands knowledge especially in relation to humor styles, resilience, and their use in a facilitative manner, as well as regarding the 3Hs in relation to populations with a sports participation background.

However, there were a number of limitations to this study. The sample used in the analyses was an easily accessible group of graduates from the faculty of sport sciences who have been in pedagogical formation training during the data collection. Therefore, the results cannot be generalized even to the department of sport sciences the participants graduated from (any department other than physical education teaching such as exercise sciences, recreation, or sports management). Although the current study provides some insights into the differences between students participating in team and individual sports and the sporting population in general, the inclusion of non-sporting participants would be useful to determine the meanings of the results providing comparisons between sporting and non-sporting populations. In addition, a limited number of participants from the study population fell within each socio-demographic and sporting variable. Future studies with similar or larger cohorts and with quota sampling regarding sporting and socio-demographic aspects would provide additional validation. Moreover, the additional validation is needed in terms of any aspect that was not provided with comparable statistics in literature as discussed above. Furthermore, there is a lack of such quests to bring together evaluations from both qualitative and quantitative analyses. Thus, qualitative analyses regarding the 3Hs, relative subjective performance, wellbeing, and the perceptions and interpretations of individuals with different backgrounds (e.g., people with or without sports participation, at higher risk of facing stress) on relevant issues would provide valuable insights. For example, previous studies on humor have led to further research on the influence of socioeconomic status, and it was difficult to compare some of our results especially regarding income, residence, and length of sports participation and specific correlations of the 3Hs due to the sparse literature.

## 5. Conclusions

This study enhances the knowledge of positive psychology in the literature by incorporating the 3Hs and social variables such as gender, age, residence, perceived income, type of sports, and the number of years’ participation in sports. The students who were sport sciences graduates scored relatively highly in all 3H instruments and their positive humor total scores were higher than the negative ones. Moreover, the results were partly in line with the so-called conventional findings in the literature. Higher scores in the use of affiliative humor were seen in females, >32 age group and respondents who reside in a megacity as well as its positive relationship with “challenge and self-commitment”. The negative humor (especially “aggressive” humor) was more often used by respondents in youngest age group, residing in towns, with higher income, and by men; negative humor also came with less resilience (self-commitment and challenge). On the other hand, resilience score was highest in students affiliated with individual sports, affiliative humor and in women. Furthermore, some of the results and complex relationships were in the furtherance of scarce literature. For example, the relationships between humor and resilience or happiness were not always positive, higher scores in the use of negative humor were seen in the respondents with higher income; men used relatively less aggressive and more affiliative humor in older age while such increase with age was not linear.

Acknowledgment of both positive and negative humor has been increasingly suggested in the literature. Our study suggests that the quests should move beyond the positive‒negative discrimination, based on the negative relationships in our results and in the literature especially between the “aggressive, self-defeating and self-enhancing” humor and PDO sub-dimensions “self-commitment and challenge”. Overall, the humor aspect in the current study, especially, became distinct for not only providing results for positive and negative humor types but also the adverse meanings revealed through their correlation with other dimensions. Moreover, the significant results in humor styles included more distinctive aspects (age, residence, and perceived income in addition to gender) while the overall significant differences in the levels of psychological hardiness were found in relation to gender, type of sports, and the number of years that the respondents had participated in these sports. Among the six sporting (2) and socio-demographic (4) variables, the gender was the only common aspect that differed significantly in all instruments (*p* < 0.05).

The discussion of the number of questions produced in this study was bounded by the scarce literature concerning combinations of traits and specific and comprehensive questions. Therefore, future research considering diverse variables (such as sporting and socio-demographic variables, or different personality traits, attitudes, or thoughts) and methodologies (such as qualitative) will continue to provide deeper understanding regarding the 3Hs.

## Figures and Tables

**Table 1:** Characteristics, by frequency and percentage, of students in pedagogical formation training.

	*N* * = 211		
		*n*/f **	% **
Gender	Female	106	50.2
	Male	105	49.8
Age	<23	40	19.0
	23–27	79	37.4
	28–32	64	30.3
	>32	28	13.3
Department	Sport Management	67	31.8
	Trainer Education	88	41.7
	Recreation	56	26.5
Sports participation	1–5 Years	39	18.5
	6–9 Years	66	31.3
	10 Years or more	106	50.2
National athlete	Yes	39	18.5
	No	172	81.5
Education (mother)	Primary school	78	37.0
	Secondary school	40	19.0
	High school	60	28.4
	University	33	15.6
Education (father)	Primary school	51	24.2
	Secondary school	39	18.5
	High school	73	34.6
	University	48	22.7
Perceived income	Low	9	4.3
	Medium	182	86.3
	High	20	9.5
Residential area	Megacity	24	11.4
	Municipality	122	57.8
	City	39	18.5
	Town	26	12.3
Sports involved	Team sports	115	54.5
	Individual sports	29	13.7
	Both team and individual sports	67	31.8

* *N*: Total number of students who participated in the study. ** The frequency (n/f) and percentage (%) of students falling in the given subgroup in the far-left column.

**Table 2:** Arithmetic mean and standard deviation values of PDO, OHQ-SF, HSQ, and sub-dimensions.

	*N* * = 211			
	$\bar{x}$ *	Sd *	Min.*	Max.*
**Psychological Hardiness (PDO)**	80.59	11.30	46.00	101.00
Challenge	28.50	4.66	14.00	35.00
Commitment	27.09	5.05	11.00	35.00
Control	24.99	3.89	15.00	33.00
**Happiness (OHQ-SF)**	25.98	4.58	13.00	35.00
**Humor Styles (HSQ)**	131.03	18.70	63.00	190.00
Affiliative	38.76	8.41	15.00	56.00
Self-enhancing	37.03	7.43	16.00	52.00
Aggressive	24.62	7.61	8.00	39.00
Self-defeating	30.61	8.32	8.00	48.00

* *N*: Total number of students who participated in the study; Arithmetic means ($\bar{x}$) from the current study; Standard deviation (Sd); Minimum (Min.) and maximum (Max) values of the instruments.

**Table 3:** The *t*-test results comparing males and females regarding PDO, OHQ-SF, HSQ, and sub-dimensions.

	*N* ** = 211					
	Gender	*N* **	$\bar{x}$ **	Sd **	*t* **	*p* *
**Psychological Hardiness (PDO)**	Female	106	82.70	10.89	2.775	0.00 *
	Male	105	78.45	11.35		
Challenge	Female	106	29.25	4.56	2.378	0.01 *
	Male	105	27.74	4.66		
Commitment	Female	106	28.22	4.63	3.330	0.00 *
	Male	105	25.96	5.22		
Control	Female	106	25.22	3.69	0.883	0.37
	Male	105	24.75	4.09		
**Happiness (OHQ-SF)**	Female	106	26.91	4.22	3.012	0.00 *
	Male	105	25.04	4.76		
**Humor Styles (HSQ)**	Female	106	132.61	16.30	1.234	0.21
	Male	105	129.43	20.81		
Affiliative	Female	106	40.05	7.34	2.266	0.02 *
	Male	105	37.45	9.21		
Self-enhancing	Female	106	38.01	6.61	1.939	0.05
	Male	105	36.04	8.08		
Aggressive	Female	106	23.94	7.03	−1.301	0.19
	Male	105	25.30	8.13		
Self-defeating	Female	106	30.59	8.06	−0.030	0.97
	Male	105	30.62	8.61		

* *p* < 0.05 = statistically significant. ** *N*: Total number of students who participated in the study; the number (*n*) of students falling in the given subgroup; Arithmetic means ($\bar{x}$); **Standard deviation (Sd), Independent *t*-test (*t*).

**Table 4:** The ANOVA results comparing age groups regarding PDO, OHQ-SF, HSQ, and sub-dimensions.

	Age Group	*n*	$\bar{x}$	Sd	*F*	*p* *
**Psychological Hardiness (PDO)**	<23	40	80.67	12.30	0.989	0.39
	23–27	79	80.48	11.59		
	28–32	64	79.31	10.57		
	>32	28	83.71	10.54		
	Total	211	80.59	11.30		
Challenge	<23	40	28.52	5.30	0.920	0.43
	23–27	79	28.32	4.33		
	28–32	64	28.12	4.76		
	>32	28	29.82	4.39		
	Total	211	28.50	4.66		
Commitment	<23	40	27.05	4.86	1.217	0.30
	23–27	79	27.05	5.45		
	28–32	64	26.50	4.72		
	>32	28	28.67	4.80		
	Total	211	27.09	5.05		
Control	<23	40	25.10	4.04	0.189	0.90
	23–27	79	25.10	3.87		
	28–32	64	24.68	3.96		
	>32	28	25.21	3.74		
	Total	211	24.99	3.89		
**Happiness (OHQ-SF)**	<23	40	26.60	4.24	0.442	0.72
	23–27	79	25.97	4.69		
	28–32	64	25.54	4.65		
	>32	28	26.14	4.74		
	Total	211	25.98	4.58		
**Humor Styles (HSQ)**	<23	40	138.42	17.06	5.199	0.00 *
	23–27	79	132.41	19.22		
	28–32	64	128.84	16.52		
	>32	28	121.57	20.14		
	Total	211	131.03	18.70		
Affiliative	<23	40	38.65	8.06	1.347	0.26
	23–27	79	39.48	8.65		
	28–32	64	37.18	8.20		
	>32	28	40.50	8.53		
	Total	211	38.76	8.41		
Self-enhancing	<23	40	38.57	6.05	3.330	0.02 *
	23–27	79	38.03	7.63		
	28–32	64	36.34	7.17		
	>32	28	33.60	8.27		
	Total	211	37.03	7.43		
Aggressive	<23	40	27.15	6.62	2.712	0.04 *
	23–27	79	23.84	7.75		
	28–32	64	25.00	7.64		
	>32	28	22.32	7.76		
	Total	211	24.62	7.61		
Self-defeating	<23	40	34.05	6.65	6.952	0.00 *
	23–27	79	31.05	8.60		
	28–32	64	30.31	7.80		
	>32	28	25.14	8.30		
	Total	211	30.61	8.32		

* *p* < 0.05 = statistically significant. ** Total number (*N*) of students who participated in the study; the number (*n*) of students falling in the given subgroup; Arithmetic means ($\bar{x}$); Standard deviation (Sd), one-way ANOVA *F*-test (*F*).

**Table 5:** The ANOVA results comparing the years participated in sports regarding PDO, OHQ-SF, HSQ, and sub-dimensions.

	Year(s) Participated in Sport	*n* **	$\bar{x}$ **	Sd **	*F*	*p* *
**Psychological Hardiness (PDO)**	1–5 years	39	84.33	10.27	2.801	0.06
	6–9 years	66	79.18	11.45		
	10 years & above	106	80.09	11.37		
	Total	211	80.59	11.30		
Challenge	1–5 years	39	29.43	4.07	0.962	0.38
	6–9 years	66	28.24	4.98		
	10 years & above	106	28.32	4.66		
	Total	211	28.50	4.66		
Commitment	1–5 years	39	28.84	5.01	3.315	0.03 *
	6–9 years	66	26.27	4.54		
	10 years & above	106	26.97	5.25		
	Total	211	27.09	5.05		
Control	1–5 years	39	26.05	3.51	1.811	0.16
	6–9 years	66	24.66	4.14		
	10 years & above	106	24.80	3.83		
	Total	211	24.99	3.89		
**Happiness (OHQ-SF)**	1–5 years	39	26.66	4.61	1.510	0.22
	6–9 years	66	25.21	4.48		
	10 years & above	106	26.21	4.62		
	Total	211	25.98	4.58		
**Humor Styles (HSQ)**	1–5 years	39	131.61	17.14	0.419	0.65
	6–9 years	66	129.28	19.75		
	10 years & above	106	131.90	18.68		
	Total	211	131.03	18.70		
Affiliative	1–5 years	39	38.66	6.81	0.127	0.88
	6–9 years	66	38.37	8.09		
	10 years & above	106	39.03	9.16		
	Total	211	38.76	8.41		
Self-enhancing	1–5 years	39	38.61	6.51	2.806	0.06
	6–9 years	66	35.36	8.73		
	10 years & above	106	37.50	6.70		
	Total	211	37.03	7.43		
Aggressive	1–5 years	39	23.46	7.46	0.590	0.55
	6–9 years	66	24.68	7.23		
	10 years & above	106	25.00	7.91		
	Total	211	24.62	7.61		
Self-defeating	1–5 years	39	30.87	8.08	0.098	0.90
	6–9 years	66	30.86	7.51		
	10 years & above	106	30.35	8.93		
	Total	211	30.61	8.32		

* *p* < 0.05 = statistically significant. ** The number (*n*) of students falling in the given subgroup; Arithmetic means ($\bar{x}$); Standard deviation (Sd), one-way ANOVA *F*-test (*F*).

**Table 6:** The ANOVA results comparing the residential areas regarding PDO, OHQ-SF, HSQ, and sub-dimensions.

	Residence	*n* **	$\bar{x}$ **	Sd **	*F* **	*p* *
**Psychological Hardiness (PDO)**	Megacity	24	84.54	11.91	2.043	0.10
	Municipality	122	80.58	11.43		
	City	39	77.51	10.71		
	Town	26	81.61	10.18		
	Total	211	80.59	11.30		
Challenge	Megacity	24	29.75	4.24	1.249	0.29
	Municipality	122	28.45	4.73		
	City	39	27.53	4.91		
	Town	26	29.03	4.25		
	Total	211	28.50	4.66		
Commitment	Megacity	24	28.29	5.06	2.620	0.05
	Municipality	122	27.33	4.95		
	City	39	25.17	5.03		
	Town	26	27.76	5.10		
	Total	211	27.09	5.05		
Control	Megacity	24	26.50	4.45	1.362	0.25
	Municipality	122	24.79	3.78		
	City	39	24.79	3.42		
	Town	26	24.80	4.40		
	Total	211	24.99	3.89		
**Happiness (OHQ-SF)**	Megacity	24	27.79	4.78	2.461	0.06
	Municipality	122	26.13	4.09		
	City	39	24.66	5.27		
	Town	26	25.61	5.10		
	Total	211	25.98	4.58		
**Humor Styles (HSQ)**	Megacity	24	141.12	19.56	3.092	0.02 *
	Municipality	122	129.97	16.90		
	City	39	131.28	18.56		
	Town	26	126.34	23.51		
	Total	211	131.05	18.70		
Affiliative	Megacity	24	41.70	9.02	3.098	0.02 *
	Municipality	122	39.36	8.07		
	City	39	35.71	8.01		
	Town	26	37.80	8.97		
	Total	211	38.76	8.41		
Self-enhancing	Megacity	24	39.33	7.86	1.053	0.37
	Municipality	122	36.75	7.00		
	City	39	36.15	8.32		
	Town	26	37.57	7.56		
	Total	211	37.03	7.43		
Aggressive	Megacity	24	25.41	7.18	3.123	0.02 *
	Municipality	122	24.00	7.18		
	City	39	27.58	7.90		
	Town	26	22.46	8.50		
	Total	211	24.63	7.60		
Self-defeating	Megacity	24	34.66	8.34	3.183	0.02*
	Municipality	122	29.86	7.86		
	City	39	31.87	8.36		
	Town	26	28.50	9.29		
	Total	211	30.61	8.32		

* *p* < 0.05 = statistically significant. ** The number (*n*) of students falling in the given subgroup; Arithmetic means ($\bar{x}$); Standard deviation (Sd), one-way ANOVA *F*-test (*F*).

**Table 7:** The ANOVA results comparing the perceived incomes regarding PDO, OHQ-SF, HSQ, and sub-dimensions.

	Perceived Income	*n* **	$\bar{x}$ **	Sd **	*F* **	*p* *
**Psychological Hardiness (PDO)**	Low	9	81.11	8.89	0.604	0.548
	Medium	182	80.85	10.93		
	High	20	77.95	15.19		
	Total	211	80.59	11.30		
Challenge	Low	9	29.77	2.38	0.907	0.405
	Medium	182	28.56	4.52		
	High	20	27.40	6.41		
	Total	211	28.50	4.66		
Commitment	Low	9	28.00	3.42	0.731	0.483
	Medium	182	27.18	4.91		
	High	20	25.90	6.77		
	Total	211	27.09	5.05		
Control	Low	9	23.33	4.52	0.976	0.379
	Medium	182	25.10	3.85		
	High	20	24.65	3.99		
	Total	211	24.99	3.89		
**Happiness (OHQ-SF)**	Low	9	26.00	5.80	0.982	0.376
	Medium	182	25.83	4.36		
	High	20	27.35	5.88		
	Total	211	25.98	4.58		
**Humor Styles (HSQ)**	Low	9	123.11	26.60	1.666	0.191
	Medium	182	130.82	18.65		
	High	20	136.45	13.97		
	Total	211	131.03	18.70		
Affiliative	Low	9	37.88	9.30	0.451	0.638
	Medium	182	38.97	8.52		
	High	20	37.20	7.09		
	Total	211	38.76	8.41		
Self-enhancing	Low	9	34.88	8.03	0.649	0.524
	Medium	182	37.25	7.46		
	High	20	36.00	6.98		
	Total	211	37.03	7.43		
Aggressive	Low	9	23.33	9.20	3.900	0.022 *
	Medium	182	24.19	7.48		
	High	20	29.05	6.93		
	Total	211	24.62	7.61		
Self-defeating	Low	9	27.00	8.76	2.816	0.062
	Medium	182	30.39	8.39		
	High	20	34.20	6.50		
	Total	211	30.61	8.32		

* *p* < 0.05 = statistically significant. ** The number (*n*) of students falling in the given subgroup; Arithmetic means ($\bar{x}$); Standard deviation (Sd), one-way ANOVA *F*-test (*F*).

**Table 8:** The ANOVA results comparing the sports participated by respondents regarding PDO, OHQ-SF, HSQ, and sub-dimensions.

	Sports	*n* **	$\bar{x}$ **	Sd **	*F* **	*p* *
**Psychological Hardiness (PDO)**	Team sports	115	78.58	11.44	4.175	0.01 *
	Individual sports	29	83.58	11.38		
	Both (team and individual)	67	82.74	10.45		
	Total	211	80.59	11.30		
Challenge	Team sports	115	27.50	4.79	6.151	0.00 *
	Individual sports	29	30.00	4.57		
	Both (team and individual)	67	29.56	4.10		
	Total	211	28.50	4.66		
Commitment	Team sports	115	26.20	5.06	4.041	0.01 *
	Individual sports	29	28.24	4.88		
	Both (team and individual)	67	28.13	4.89		
	Total	211	27.09	5.05		
Control	Team sports	115	24.86	3.98	0.180	0.83
	Individual sports	29	25.34	4.05		
	Both (team and individual)	67	25.04	3.71		
	Total	211	24.99	3.89		
**Happiness (OHQ-SF)**	Team sports	115	25.73	4.43	0.392	0.67
	Individual sports	29	26.34	4.36		
	Both (team and individual)	67	26.26	4.96		
	Total	211	25.98	4.58		
**Humor Styles (HSQ)**	Team sports	115	132.01	19.28	1.139	0.32
	Individual sports	29	133.58	20.68		
	Both (team and individual)	67	128.31	16.65		
	Total	211	131.05	18.70		
Affiliative	Team sports	115	38.42	8.59	0.234	0.79
	Individual sports	29	39.48	8.79		
	Both (team and individual)	67	39.04	8.00		
	Total	211	38.76	8.41		
Self-enhancing	Team sports	115	37.09	7.87	0.183	0.83
	Individual sports	29	37.65	7.62		
	Both (team and individual)	67	36.67	6.61		
	Total	211	37.03	7.43		
Aggressive	Team sports	115	25.13	7.90	0.802	0.45
	Individual sports	29	24.93	7.22		
	Both (team and individual)	67	23.67	7.25		
	Total	211	24.63	7.60		
Self-defeating	Team sports	115	31.36	8.61	2.039	0.13
	Individual sports	29	31.51	8.31		
	Both (team and individual)	67	28.92	7.65		
	Total	211	30.61	8.32		

* *p* < 0.05 = statistically significant. ** The number (*n*) of students falling in the given subgroup; Arithmetic means ($\bar{x}$); Standard deviation (Sd), one-way ANOVA *F*-test (*F*).

**Table 9:** Common or different aspects regarding significant results.

Instruments	Common: Significantly Different Results in Three Instruments	Different: Significantly Differ in Particular Instrument(s) Only and Absent in Other(s)
Happiness (OHQ-SF)	Gender	
Hardiness/Resilience (PDO)		length of years participated in these sports, type of sports
Humor (HSQ)		age, residence & perceived income

## Appendix A

**Table A1:** The Alpar (2014) qualification regarding correlation coefficient [29].

r-Value	Qualification
0.00–0.19	No or negligible relationship
0.20–0.39	Weak relationship
0.40–0.69	Moderate
0.70–0.89	Strong relationship
0.90–1.00	Very strong relationship
